# How to Use TeleSimBox “Off the Shelf” to Connect Remote Content Experts With In-Person Simulation Participants

**DOI:** 10.7759/cureus.16317

**Published:** 2021-07-11

**Authors:** Elizabeth Sanseau, Robert Cameron Sooby, Maybelle Kou, Marc Auerbach, Khoon-Yen Tay

**Affiliations:** 1 General Pediatrics/Emergency Medicine, Children's Hospital of Philadelphia, Philadelphia, USA; 2 Emergency Medicine, Jefferson Health Northeast, Philadelphia, USA; 3 Emergency Department, Inova Children's Hospital, Falls Church, USA; 4 Department of Pediatrics, Section of Pediatric Emergency Medicine, Yale University, New Haven, USA; 5 Emergency Medicine, Children's Hospital of Philadelphia, Philidelphia, USA

**Keywords:** emergency medicine education, simulation, physical distancing, telesimulation, open access medical education

## Abstract

In this technical report, we describe how to use TeleSimBox to run a remotely facilitated simulation to connect the facilitator with learners at a distant site. This method was developed to comply with safety measures imposed during the coronavirus disease-19 (COVID-19) pandemic to reduce the risk of viral exposure and transmission. Here, we present one example where a telesimulation naïve facilitator was trained as an in-person facilitator to enable the in-situ medical student and resident learners to participate in a pediatric emergency simulation exercise remotely guided by an off-site content expert. The case of neonatal shock was run five times during a half-day emergency department (ED) educational program with one to four participants per session. 14/15 (93%) participants completed evaluations and felt that the simulation met the case learning objectives and that connecting with the remote facilitator was useful for their learning. Feedback from the one newly trained in-person facilitator was that the tool was easy to learn how to use quickly, and the process of connecting with a remote expert was worthwhile for learners. To grab this web-based toolkit off the proverbial shelf and successfully run a telesimulation session from start to finish took approximately one hour; 20 minutes were spent in preparation the day prior and 40 minutes to set up and run the simulations the day of. We believe that this is a low-cost, efficient, and perceived to be an effective method to connect remotely located content experts and learners to engage in a simulation-based education activity when access to in-person resources and personnel is limited.

## Introduction

Simulation is defined as a technique that creates a situation or environment to allow persons to experience a representation of a real event for the purpose of practice, learning, evaluation, testing or to gain an understanding of systems or human actions [1]. Remote simulation is defined as “simulation performed with either the facilitator, learners, or both, in an offsite location separate from other members to complete educational or assessment activities. Facilitation and assessment can be performed either synchronously or asynchronously using video or web conferencing tools” [1].

The American College of Emergency Physicians (ACEP) SimBox is a free, openly available web-based simulation education platform that was initially designed to provide a way for a simulation-naïve facilitator to be able to conduct their own simulation exercises to improve pediatric readiness without access to the resources of a simulation center or expert [2]. The peer-reviewed content on the SimBox website is designed by physicians, grounded in simulation and adult learning theory, and thus far has been used over 6,000 times across the globe [3,4]. Due to initial feedback by users requesting the option to teleconnect with a pediatric specialist to help use the SimBox, accelerated by the need for telesimulation tools during the coronavirus disease-19 (COVID-19) pandemic, creators adapted the website content for remote simulation as the “TeleSimBox,” using teledebriefing [5] and telementoring [6] concepts.

The COVID-19 pandemic has challenged medical education at all levels, forcing educators to adapt didactic modalities to reduce exposures to learners, facilitators, and content experts [7]. Organizers of an annual in-person education fair hosted by an Emergency Medicine Residency adapted their program agenda to adhere to the most up-to-date COVID-19 safety regulations as of April 2021 [8]. To remain in compliance, masked in-person learner groups were limited to a maximum of four per room plus one instructor. The organizer in charge of the pediatric portion of the program requested telesimulation options offered by colleagues at a nearby children’s hospital simulation center that in prior years had offered in-person simulation training. This organizer, a third-year adult Emergency Medicine Resident who served as the in-person newly trained co-facilitator for the pediatric simulation case, was trained in how to use the TeleSimBox resources [2].

In this technical report, we outline how to conduct a simulated medical scenario with a maximum of four in-person learners and one in-person facilitator who remotely connects to an off-site educator using the TeleSimBox tool. The scenario of neonatal shock was selected by the planning committee, as the case is a low-frequency, high-stakes, anxiety-inducing, pediatric scenario that has been demonstrated to be effective to teach medical and teamwork learning objectives to physicians-in-training [9]. The half-an-hour remotely facilitated simulation session was offered as an option for learners to rotate through during a half-day procedure and simulation fair hosted by the Emergency Medicine Residency Program in their own emergency department (ED). The newly trained in-person facilitator successfully enabled in-person medical students and non-pediatric residents specializing in family, internal and emergency medicine to actively engage in the simulation session by teleconnecting to an off-site facilitator. This remote facilitator is co-creator of the TeleSimBox tool, is an experienced user of the resources, and specializes in pediatric emergency medicine. This report outlines the steps of how the session was planned and enacted.

## Technical report

Pre-simulation preparation

**Figure 1:**
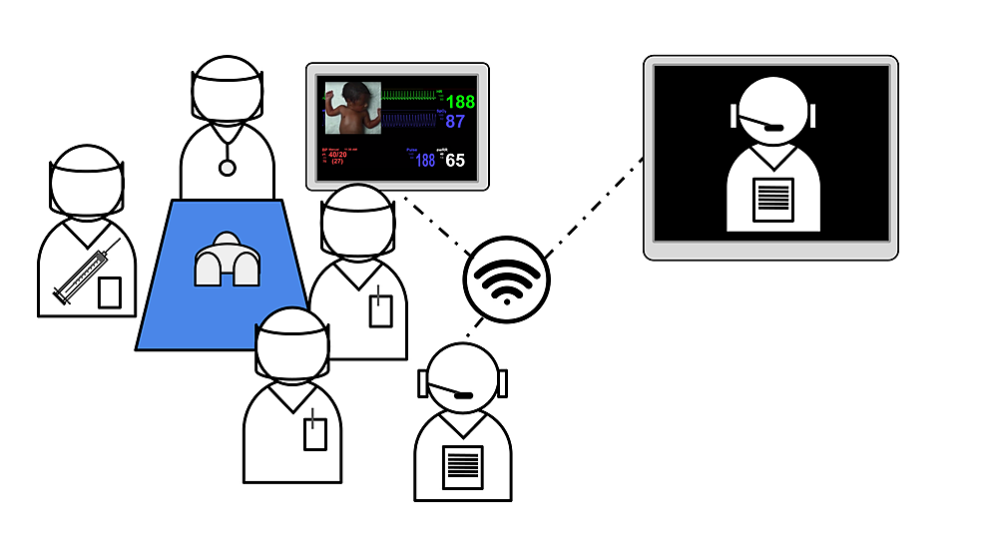
Pictogram of remotely guided simulation setup. Courtesy of Dr. Maybelle Kou, MD.

The in-person facilitator prepared for and co-facilitated the simulation from the same space as the learners, enabling the teleconnected activity to happen (Figure 1). The session was run in-situ in the real ED rooms, with the pediatric equipment and supplies typically available. No special simulation center, personnel, technology, or extra equipment was used. The only additional supplies to set up were a simple baby doll, baby warmer, and two laptop computers with web cameras optimally positioned for remote facilitation.

**Figure 2:**
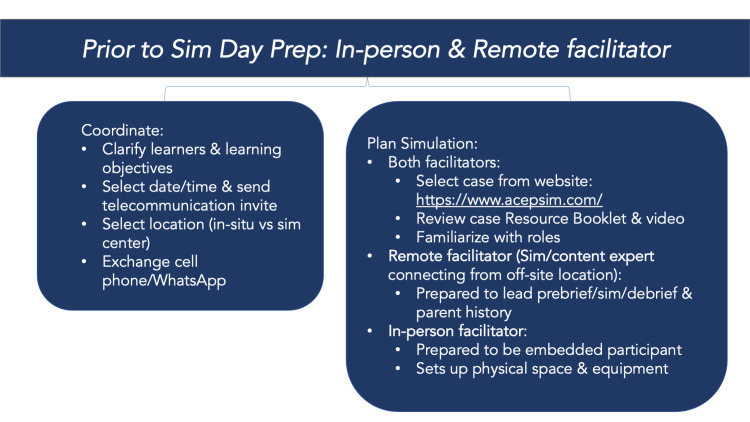
Preparation for facilitators: prior to simulation.

The facilitators met for approximately 20 minutes the day before to prepare for the session. During this time, they selected a case, reviewed the Resource Booklet, video, and Quick Guide from the TeleSimBox website [2], coordinated the learning objectives for the specific learners, exchanged cell phone contact information, and created a teleconferencing link for the session. They clarified if the in-person learners and in-person facilitators would meet in a teaching room or in-situ in the ED. If the latter, the in-person facilitator was encouraged to communicate with the charge nurse to maintain situational awareness and obtain appropriate permission if needed. After this meeting, the in-person facilitator focused on familiarizing with the role of the embedded participant. Specifically, they prepared for how to respond to learners when requesting an initial assessment of the patient, access, medications, fluids, laboratories or imaging, airway maneuvers, and reassessment throughout the case. Before the simulation session, the remote facilitator also closely reviewed the Resource Booklet, including the parental history. Both facilitators reviewed the case video and were prepared to run the debrief using prompts and education resources included in the Resource Booklet (Figure 2).

Day-of-simulation details

**Figure 3:**
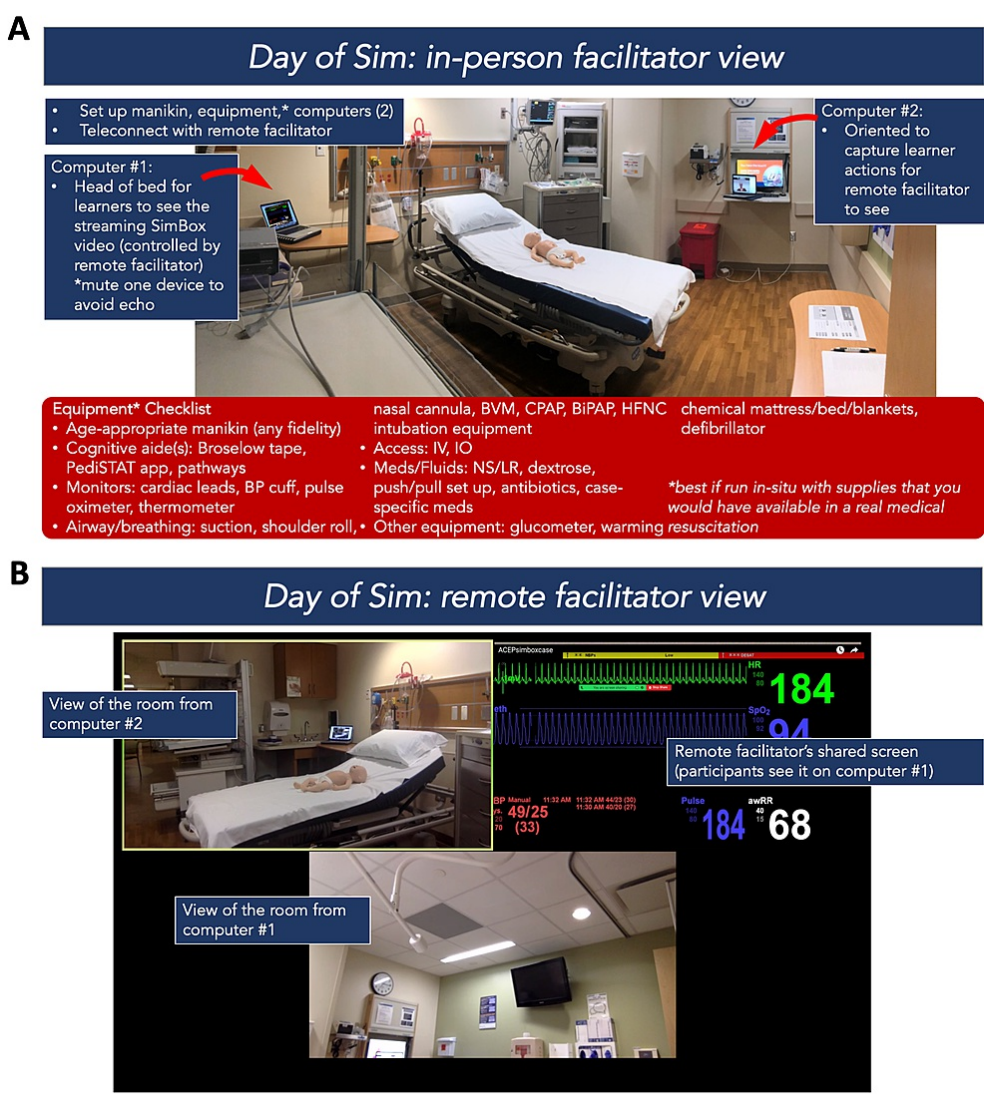
Preparation for facilitators: day of simulation. (A) In-person facilitator view and (B) remote facilitator view.

**Figure 4:**
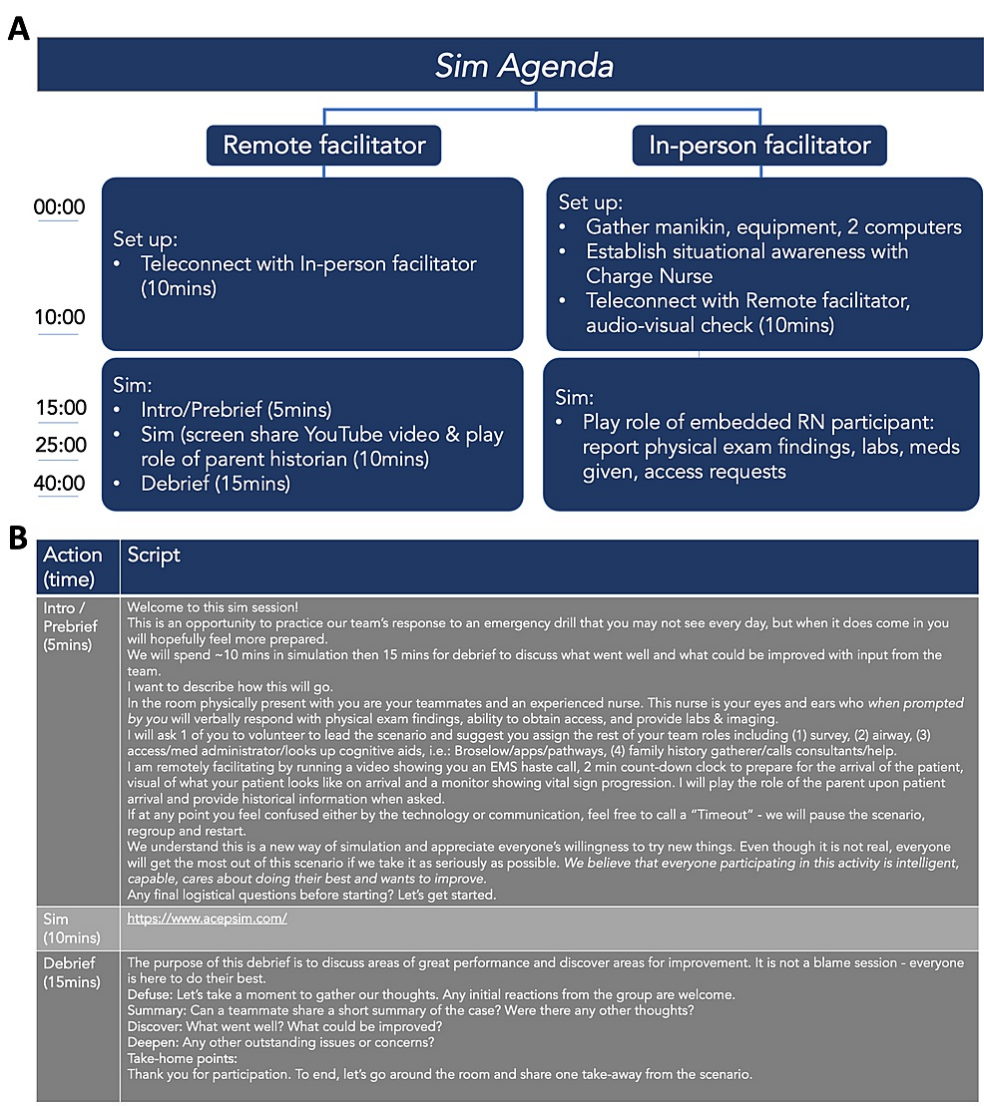
(A) Agenda for remote and in-person facilitator and (B) script for a remote facilitator for introduction, prebrief, simulation and debrief.

The co-facilitators teleconnected via the meeting link 10 minutes prior to the students’ arrival to optimize the audio-visuals, troubleshoot any connection issues or camera views, and review roles. During the simulation, they communicated via text message on their cell phones about any logistics that came up, including the flow of timing and audio-visual adjustments when needed. Thirty minutes were allotted to run each simulation (including the prebrief, simulation, and debrief). In standard simulation language, the prebrief is defined as an activity immediately preceding the start of a simulation drill where the participants receive essential information about the scenario, including relevant patient background information [1]. The TeleSimBox prebriefing toolkit includes scripted tips to establish psychological safety in simulation-based education adapted from the Center For Medical Simulation at Boston Children’s Hospital [10]. The debrief is defined as a formal, collaborative, reflective process that a facilitator leads following a simulation experience [1]. Here, the remote facilitator used scripts to ensure adherence to basic simulation prebrief and debrief components and maintain efficiency and consistency in repeated sessions. For this half-day education fair, the same 30-minute simulation session of neonatal shock was repeated five times with groups composed of one to four medical student and resident trainee learner groups; a total of 15 learners participated (Figures 3 and 4).

TeleSimBox simulation technique

**Figure 5:**
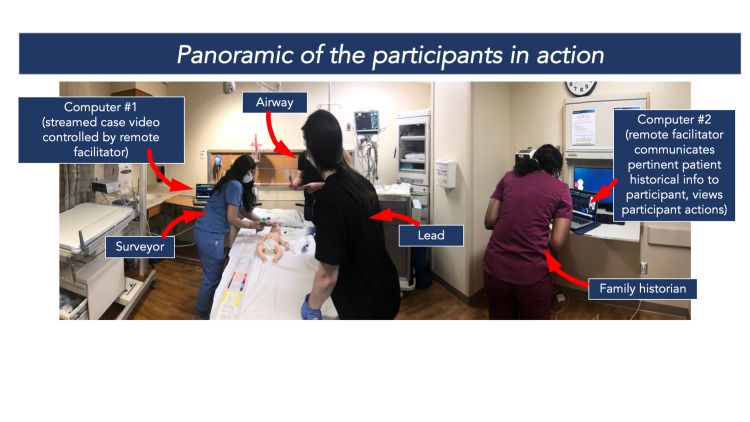
Participants in action, view from the in-person facilitator. Courtesy of Dr. Robert Cameron Sooby, DO.

The session started with an introduction and prebrief by the remote facilitator. The simulation began by playing the TeleSimBox YouTube^©^ video accessible through the website including a transport team call into the hospital, a two-minute countdown clock, a visual of an ill-appearing patient, and a pre-recorded beeping monitor with vital signs that progressed from poor to improved over a 10-minute span. The remote facilitator observed the actions of the participants using the teleconference gallery view. They also played the role of the parent, providing pertinent patient historical information when asked by the learners. The in-person facilitator responded to participant requests for the initial patient assessment, ability to get intravenous versus intraosseous access, and re-examination following key airway, breathing, circulatory, and disease-specific medical interventions. After 10 minutes, the remote facilitator concluded the scenario by asking a participant to sign the patient out to the transfer team prior to proceeding to a 15-minute simulation debrief. Immediately following the simulation, learners were asked to complete a survey before heading to the next station (Figure 5).

COVID-19 safety compliance

In compliance with COVID-19 safety guidelines, we limited the in-person class size to four students and one instructor. All students and instructors were previously vaccinated with the COVID vaccine, wore masks, were screened for illness, and practiced proper hand hygiene. The groups rotated through different procedure and simulation stations throughout this half-day education fair hosted in-situ in the ED, including this remotely facilitated pediatric simulation station. All equipment was wiped down with alcohol-based wipes between each use. While it is admittedly very difficult to maintain safe social distancing during hands-on simulation, each student had a different role and assumed positions around the manikin >3 feet apart from each other. Due to limitations in the number of in-person people allowed, the remote facilitator teleconnected into the room to remotely facilitate the drill.

Evaluation

We collected post-simulation surveys to capture learner perceptions on the effectiveness of this remote simulation method. Learners were initially asked how prepared they felt to respond to a neonate in shock in their ED in general. They were subsequently asked specific questions about the simulation using a five-point Likert scale (one being strongly disagree, five being strongly agree): (i) The exercise met the teamwork/communication learning objectives: verbally assemble the necessary staff, equipment, and resources to care for a neonate in shock. (2) The exercise met the teamwork/communication learning objectives: demonstrate effective teamwork and communication (i.e., sharing mental model, directed orders, closed-loop communication). (iii) The exercise met the family-centered care learning objectives: obtain an appropriate history from the family member (SAMPLE). (iv) The exercise met the family-centered care learning objectives: address family concerns, update on the care. (v) The exercise met the medical knowledge learning objectives: verbalize the initial management of an acutely ill pediatric patient (ABCs). (vi) The exercise met the medical knowledge learning objectives: recognize a neonate in shock (a neonate with abnormal vital signs and concerning clinical exam). (vii) The exercise met the medical knowledge learning objectives: verbalize the first-line therapeutic interventions for shock (fluids, antibiotics, temperature, and glucose control). (viii) The debrief with a remote facilitator fostered reflective learning. (ix) The facilitator(s) prompts in the debrief were relevant and helped keep the conversation focused on the case learning objectives.

Learners were finally asked to respond to three open-ended questions: what changes would you make to improve this exercise? What did you like about this remotely facilitated simulation? Would you like remotely facilitated simulated experiences in the future?

In addition, we conducted a semi-structured interview with the newly trained in-person facilitator to assess the resources, including time and materials, necessary to train co-facilitators to conduct this type of simulation exercise.

## Discussion

Learner demographics

A total of 15 learners rotated through this simulation station, with groups ranging from one to four participants per group, divided between five sessions. Fourteen learners completed a post-simulation survey, for a 14/15 (93%) response rate. Learners who responded included 3 medical students and 11 residents. Residents were from emergency medicine, family medicine, emergency medicine/internal medicine, and emergency medicine/family medicine residency programs. Medical students were either in their third or fourth year of training. Over 50% of learners had more than 10 prior in-person simulations, 14.3% had between 6 and 10, and 35.7% had less than 5; 100% of learners had fewer than five remotely facilitated simulation experiences.

Survey results

When it came to preparedness, there was a wide range of responses. Pre-simulation, the average learner saw themselves as somewhere in the middle between unprepared and prepared to manage a neonate in shock. This was likely due to the wide range of learner experiences, ranging from medical student to second-year emergency medicine residents. A review of the post-simulation survey responses gathered from the participants on their perception of learning objective attainment (on a Likert Scale from one-strongly disagree to five-strongly agree) resulted in calculated means all greater than four. The two teamwork and communication questions scored 4.57 and 4.71. The two family-centered care learning objectives scored 4.50 and 4.57. The three medical knowledge objectives each scored 4.86. All 14 respondents strongly agreed (5.0) that the debrief learning objectives were met (Table 1).

**Table 1:** Post-course evaluation of learners: teamwork/communication, family-centered care, medical knowledge, and debrief

Likert Scale: 1 - strongly disagree, 2 - disagree, 3 - neutral, 4 - agree, 5 - strongly agree				
Statement	Participants Med students (n); residents (n)	Mean (standard deviation)	Median	Range
The exercise met the teamwork/communication learning objectives: verbally assemble the necessary staff, equipment, and resources to care for a neonate in shock.	Med students: 3; residents: 11	4.57 (0.65)	5	3 - 5
The exercise met the teamwork/communication learning objectives: demonstrate effective teamwork and communication (i.e.: sharing mental model, directed orders, closed-loop communication).	Med students: 3; residents: 11	4.71 (0.47)	5	4 - 5
The exercise met the family-centered care learning objectives: obtain an appropriate history from the family member (SAMPLE).	Med students: 3; residents: 11	4.50 (0.65)	5	3 - 5
The exercise met the family-centered care learning objectives: address family concerns, update on the care.	Med students: 3; residents: 11	4.57 (0.51)	5	4 - 5
The exercise met the medical knowledge learning objectives: verbalize the initial management of an acutely ill pediatric patient (ABCs).	Med students: 3; residents: 11	4.86 (0.36)	5	4 - 5
The exercise met the medical knowledge learning objectives: recognize a neonate in shock (a neonate with abnormal vital signs and concerning clinical exam).	Med students: 3; residents: 11	4.86 (0.36)	5	4 - 5
The exercise met the medical knowledge learning objectives: verbalize the first-line therapeutic interventions for shock (fluids, antibiotics, temperature, and glucose control).	Med students: 3; residents: 11	4.86 (0.36)	5	4 - 5
The debrief with a remote facilitator fostered reflective learning.	Med students: 3; residents: 11	5.0 (0)	5	5
The facilitator(s) prompts in the debrief were relevant and helped keep the conversation focused on the case learning objectives.	Med students: 3; residents: 11	5.0 (0)	5	5

In the free response, learners suggested to increase the duration of the simulation and to have a separate facilitator serve as the family member providing the patient history. The latter would allow the remote facilitator to better observe the participants in action with less distraction. When asked what they liked about this remotely facilitated simulation in the free-response section, a plurality stated the debrief. Others liked the scenario chosen, saying that it was “realistic” and “interactive.” Some liked how the session was “concise” and “efficient.” One even reported that the simulation felt more real having a remote facilitator rather than just an in-person facilitator. All 14 respondents stated they would like more blended in-person with remote facilitated simulated experience in the future. Other scenarios suggested included: pediatric trauma, pediatric cardiac arrest, and pediatric respiratory failure specifically for bronchiolitis and asthma.

In-person facilitator feedback

A semi-structured interview with the newly trained in-person facilitator was conducted to obtain demographic and resource data. The in-person facilitator was a third-year Emergency Medicine resident who was experienced in simulation as a learner, less so as a facilitator, and naïve with telesimulation. Prior to this educational session, he had participated as a learner in more than 10 in-person simulations, facilitated under five in-person simulations, participated as a learner in less than five remotely facilitated simulations, and facilitated zero remote simulations. To prepare for this educational session, he spent approximately 30 minutes reviewing the TeleSimBox Quick Guide and watching part of the sample video, both accessible on the TeleSimBox website in addition to meeting one-on-one for a tutorial with a core member of the TeleSimBox team who was also the remote facilitator for this trial. He opted not to read the resource booklet, watch the case video, or conduct a practice session because he had met one-on-one with a TeleSimBox core team member already.

Preparation of the simulation room, which included bringing equipment (baby doll manikin, baby warmer, two computers) and connecting to the remote facilitator via the teleconferencing tool Zoom^©^, took approximately 10 minutes the day of the simulation. Each simulation scenario itself took approximately 20-30 minutes. In total, this in-situ facilitator spent approximately one hour to familiarize, prepare, and enable the remotely facilitated simulation didactic to occur. He described the TeleSimBox as “very easy to co-facilitate as the in-person facilitator.” His main suggestion was to make the list of the necessary equipment and supplies to run the simulation more obviously accessible on TeleSimBox preparatory materials. He reported: “I found the simulation very easy to use and co-facilitate. I believe anyone can use it with ease. The debriefing experience was excellent, and I know many of the participants hold the same sentiment. Too many times debriefing sessions are too short. Not this experience. It provided real-time identification of strengths and weaknesses, burning into their heads what they missed or got wrong, so they would not forget next time. I believe the designated amount of time for SIM and debriefing to be sufficient.”

Limitations

This technical report is meant to outline one quick, easy, and low-cost method to provide a blended in-person and remotely co-facilitated simulation activity. We provide feedback from a small convenience sample of resident and medical student users who participated during a half-day emergency medicine education day. We also provide feedback from the newly trained in-person co-facilitator. We do not provide an assessment of the remote facilitator, who is a content expert and co-designer of the TeleSimBox tool. This is not meant to be a comprehensive evaluation of the TeleSimBox tool. We did not assess actual participant learning with pre- and post-simulation tests but instead evaluated the learners’ experience in a post-simulation survey and the newly trained facilitator’s experience in a semi-structured interview. While we did not have any technical issues during this half-day simulation day, this is always a risk when running a didactic exercise relying on computer devices remotely connecting via the internet. In terms of safety limitations, while we made efforts to comply with safe distancing restrictions to reduce the risk of viral spread, participants did not always stay more than 3 feet apart during the simulated exercise and when gathered around the computer to participate in the debrief. Future sessions might consider placing taped markings on the floors to restrict where participants can stand. The alternative to this session would have been cancelling the pediatric emergency simulation training at this education fair. We believe this remotely-guided simulation method successfully met the learning objectives for these non-pediatric medical students and resident trainees with limited risk of COVID-19 transmission. We did not test if COVID-19 cases were transmitted during the training session, however, we have not had any cases reported in this vaccinated and masked user group.

Next steps

The TeleSimBox core team regularly updates the website content. Following this trial, some changes were implemented. Based on the in-person facilitator feedback, the TeleSimBox Quick Guide on the website now includes a list of the supplies needed for the in-person facilitator to set up and is highlighted in this report (Figure 3). Based on learner feedback, we recommend that the role of the parent not be played by the remote facilitator, but instead, by either another facilitator (either in-person or remotely connecting, if/when available) or have the in-person facilitator provide the history in a creative way. For example, the in-person facilitator might relay the main patient historical points that medics reported from the scene saying that the parent is “en route” to the hospital. The case Resource Booklets and videos have been updated on the TeleSimBox website. To help novice simulation facilitators use this simulation resource toolkit, the video now includes pre-recorded prebrief and debrief sections. In essence, the facilitator now simply starts the video which takes the users on a self-guided simulation exercise with even less preparation by the facilitator. While we anticipate that the updated version with these recorded prebrief and debriefing resources will likely take longer to run than the method outlined in this report, we think this will make the tool easier for simulation-naïve facilitators to use. Those facilitators who do not wish to use the pre-recordings can simply skip the recorded prebrief and debrief sections and use the method outlined here and resources on the website to guide the didactic. We have yet to receive formal feedback on this new iteration and welcome future users, comments, and research with the tool. Future steps are to compare this simulation teaching method with a standard in-person course, either from previous participants of a standard course or prospectively after the pandemic is controlled.

## Conclusions

Medical students and non-pediatric resident trainees have limited exposure to managing acutely ill pediatric patients during their training, with even fewer opportunities for exposure during the pandemic given the imposed restrictions to in-person clinical rotations and didactics. This technical report outlines a step-by-step approach describing how to tele-connect a content expert to an in-person group of learners and newly trained in-person facilitators who otherwise would not have access to this type of hands-on training. Feedback from the 14 medical student and resident physician learner respondents and one in-person newly trained facilitator indicates that the sessions were easily and successfully implemented without technology failures, the scenario’s medical and teamwork learning objectives were met, and the learners felt that the debrief with the remotely connecting expert was useful for their learning.

Based on this small example, we believe this is a quick and easy-to-use option for this type of learning, useful for pandemic times and beyond. The TeleSimBox tool is a low-cost way to bridge geographic and resource gaps by connecting experts in pediatric emergency medicine and simulation with community providers and trainees; the ultimate intention is to optimize provider confidence, competence, and patient outcomes.

## References

[1] (2021). The second edition (2.1) of the Healthcare Simulation Dictionary. https://www.ssih.org/Dictionary.

[2] (2021). ACEP TeleSimBox: Leveraging technology for remote learning. https://www.acepsim.com/.

[3] Zigmont JJ, Kappus LJ, Sudikoff SN (2011). Theoretical foundations of learning through simulation. Semin Perinatol.

[4] Taylor DC, Hamdy H (2013). Adult learning theories: implications for learning and teaching in medical education: AMEE Guide No. 83. Med Teach.

[5] Cheng A, Kolbe M, Grant V (2020). A practical guide to virtual debriefings: communities of inquiry perspective. Adv Simul (Lond).

[6] Gross IT, Whitfill T, Auzina L, Auerbach M, Balmaks R (2021). Telementoring for remote simulation instructor training and faculty development using telesimulation. BMJ Simul Technol Enhanced Learn.

[7] Rose S (2020). Medical student education in the time of COVID-19. JAMA.

[8] (2021). AMA guiding principles to protect learners responding to COVID-19. https://www.ama-assn.org/delivering-care/public-health/ama-guiding-principles-protect-learners-responding-covid-19.

[9] Sanseau E, Lavoie M, Tay KY (2021). TeleSimBox: a perceived effective alternative for experiential learning for medical student education with social distancing requirements. AEM Educ Train.

[10] (2021). Center For Medical Simulation, Boston Children’s Hospital. https://harvardmedsim.org/.

